# The effects of vasopressor choice on renal outcomes in septic shock: a systematic review of randomised trials as a guide for future research

**DOI:** 10.1186/s13054-025-05573-7

**Published:** 2025-10-02

**Authors:** Rory McDonald, Michael Burns, Adrian Wong, Carolyn Smith, Marlies Ostermann, Sam Hutchings

**Affiliations:** 1 Department of Inflammation Biology, School of Immunology and Microbial Sciences, King’s College London, London, UK; 2 Academic Department of Military Anaesthesia and Critical Care, Royal Centre for Defence Medicine, Birmingham, UK; 3 Department of Critical Care, King’s College Hospital NHS Foundation Trust, London, UK; 4 Department of Critical Care, University Hospitals Bristol and Weston NHS Foundation Trust, Bristol, UK; 5 Bodleian Health Care Libraries, University of Oxford, Oxford, UK; 6 Department of Critical Care, Guy’s & St Thomas’ NHS Foundation Trust, London, UK

**Keywords:** Septic shock, Vasopressors, Acute kidney injury, Renal outcomes

## Abstract

**Background:**

Patients with septic shock are high risk for developing acute kidney injury (AKI), with its associated morbidity. This systematic review assessed the evidence for an effect on renal outcomes from choice of vasopressor.

**Methods:**

Searches were conducted on Medline, Embase, Cochrane Central, congress abstracts and trial registries. The search strategy included septic shock, vasopressor agents and renal impairment. Inclusion criteria were non-crossover randomised controlled trials of adult septic shock comparing individual or combinations of vasopressors and placebo controlled trials. Primary outcome was the incidence of AKI in study participants. Secondary outcomes were AKI duration, renal replacement therapy (RRT) rate, RRT duration, renal failure free days, requirement for long term RRT and Major Adverse Kidney Events (MAKE) at 30 and 90 days.

**Results:**

A total of 4259 patients, from 17 studies, were included. Vasopressin and terlipressin studies predominated. In 8 studies reporting AKI rate, no effect was seen relating to vasopressor choice. RRT rate was the most reported secondary outcome. Of five studies that investigated the role of vasopressin, only one showed significant benefit. Alongside limited reporting, no conclusive benefit was demonstrated in other secondary outcomes. No studies reported requirement for long term RRT, MAKE 30 or 90.

**Conclusions:**

This is the first systematic review focussed on renal outcomes with differential vasopressor therapy in septic shock. It illustrates the paucity of evidence supporting a particular vasopressor. Also highlighted are problems of population and study heterogeneity, as well as the focus on RRT as a proxy for renal outcomes. Standardised renal outcome reporting, large and appropriately powered trials and focussed sub-population studies are required to further inform renal focussed vasopressor research and practice.

**Trial registration:**

This systematic review was prospectively registered on PROSPERO (CRD42023481778).

**Supplementary Information:**

The online version contains supplementary material available at 10.1186/s13054-025-05573-7.

## Background

Sepsis is defined as life-threatening organ dysfunction caused by a dysregulated host response to infection [1]. Amongst patients with sepsis the most severely affected will be in septic shock, defined as a requirement for vasopressor therapy and a raised serum lactate [1]. The mortality rate in this patient population is in the region of 35–40% [2, 3]. Although incompletely understood in terms of mechanism, patients with septic shock are at high risk for the development of acute kidney injury (AKI) [4]. This syndrome accounts for almost half of all cases of AKI seen in the critically ill, and results in a further increase in mortality of up to 70% [5, 6]. Understanding specific risk factors for AKI, as well as possible preventative measures, is therefore of vital importance in the management of critically ill septic patients.

Vasopressors are the mainstay of vasodilatory shock management. In addition to classically used catecholamines, non-adrenergic vasopressors are commercially available that target either the vasopressin pathway or renin-angiotensin system (RAS). In septic shock, the Surviving Sepsis Campaign (SSC) recommends intravenous crystalloid resuscitation as well as norepinephrine to maintain mean arterial pressure (MAP) targets. Where norepinephrine alone is insufficient, it is suggested that vasopressin be added as a second agent [7]. However, the SSC describe the evidence underpinning these recommendations as high quality only when first line norepinephrine is compared to dopamine. When compared to other catecholamines and non-adrenergic vasopressors the evidence is of moderate, low or very low quality. In addition, the evidence underpinning the recommendation of vasopressin, where a second agent is required, is of moderate quality. This paucity of good quality evidence perhaps contributes to the apparent variation in standards of care relating to vasopressors [2, 8]. In addition, these guidelines do not fully consider cost, which can be important [9].

To date, most systematic reviews investigating the effects of different vasopressors in septic shock have focussed on mortality [10–18]. These reviews frequently target particular vasopressors, and many have been judged as low quality using the AMSTAR 2 tool [19, 20]. Despite their clinical importance, renal outcomes are often secondary considerations or not reported. This is surprising as experimental evidence suggests that different vasopressors produce varying effects on renal perfusion and subsequent injury. Where reviews are designed with renal focussed primary outcomes, these often include heterogeneous shock populations [21, 22]. A summary of related systematic reviews is shown in Table 1. Specific to septic shock, the relationship between vasopressors and renal outcomes has, therefore, been incompletely studied.

**Table 1:** Summary of related systematic reviews

Study (year)	Design	P	Intervention	Comparator	Primary outcome	Renal secondary outcomes	Study types	Number of studies included	Number of patients included	Renal outcome results	Comments
Serpa et al. [17] (2012)	SR, MA	VS	V, T	Catecholamine vasopressor	Mortality		RCTs	9	998		No renal outcomes
Avni et al. [10] (2015)	SR, MA	SS	Any vasopressor or combination	Different vasopressor or combination, placebo, no vasopressor	Mortality	UO	RCTs, (including crossover)	32	3544	Higher UO with N (vs D)	
Ye et al. [11] (2023)	SR, MA	SS	Early vasopressor	Late vasopressors	Mortality	AKI rate, RRT rate	RCTs, observational studies	23	25,721	Lower AKI & RRT rate with early vasopressors	3 and 7 studies included in AKI and RRT rate evaluation, respectively
Sedhai et al. [12] (2022)	SR, MA	SS	First line V	First line N	Mortality	UO, RRT rate	RCTs, observational studies	8	2182*	No difference in UO, lower RRT rate with first line V	2 studies included for renal outcome evaluation, dominated by Gordon et al. [41]
Backer et al. [13] (2012)	SR, MA	SS	D	N	Mortality	RRT free days	RTs, observational studies	11	2768	Insufficient data for evaluation	
Huang et al. [14] (2019)	SR, MA	SS	T	N	Mortality	UO, SCr	RCTs	6	756	No difference	
Huang et al. [15] (2021)	SR, MA	SS	V within 6 h	No V within 6 h or V after 6 h	Mortality	RRT rate	RCTs, observational studies	5	788	Lower RRT rate with V within 6 h	3 studies included for renal outcome evaluation
Belletti et al. [16] (2015)	SR, MA	VS	Non-catecholamine vasopressors	N, D, P	Mortality		RCTs	20	1608		No renal outcomes
Nagendran et al. [18] (2019)	IPD MA	SS	V	Other vasoactive comparator	Mortality, number of SAEs	RRT rate, RRT duration, renal failure free days	RCTs (non-crossover)	4	1453	Lower RRT rate with V. No difference in RRT duration or renal failure free days	Results not significant when assessed using random effects model
Vernon-Elliot et al. [21] (2024)* ∞*	SR	VS	Non-catecholamine vasopressors	Catecholamine vasopressors	AKI rate, RRT rate/duration/liberation, CCR, SCr, UO, BUN, GFR, other biomarkers		RCTs, observational studies	49	8274	Lower RRT rate & improved liberation with non-catecholamines. No difference in other outcomes	Qualitative analysis. 3 studies for RRT rate outcome assessment (all V). 1 study with RRT liberation outcome
Nedel et al. [22] (2019)	SR, MA	DS	V	Other vasopressors	RRT rate, AKI rate, AKI free days		RCTs	17^**^	2833	Lower RRT & AKI with V. AKI free days results mixed	In septic shock subpopulation no difference in RRT (4 studies) or AKI (6 studies) with V

*P* Population, *S* studies, *SR* systematic review, *MA* meta-analysis, *IPD* individual patient data, *SS* septic shock, *VS* vasodilatory shock, *DS* distributive shock, *V* vasopressin, *D* dopamine, *T* terlipressin, *N* norepinephrine, *P* placebo, *AKI* acute kidney injury, *RRT* renal replacement therapy, *CCR* creatinine clearance rate, *SCr* serum creatinine, *BUN* blood urea nitrogen, *GFR* glomerular filtration rate, *SAEs* serious adverse events, *UO* urine output, *RCT* randomised controlled trials, *RT* randomised trials

^*^Propensity matching study. ∞ Also includes pre-clinical shock model component. ^**^Only 11 studies (2691 individuals) included in MA

There is currently great interest in individually tailored vasopressor therapy, particularly for those at risk of adverse renal outcomes [24, 25]. However, in order to achieve this therapeutic aim, greater understanding of the relationship between the arsenal of vasopressors and renal injury is required [26]. This is in addition to improved phenotypic classification of patients, identifying those likely to benefit from particular interventions or at risk of adverse events and outcomes [27].

The objectives of this systematic review were to determine whether the choice of vasopressor agent affects both short- and longer-term renal outcomes in adult patients with septic shock, and, through appraisal of the evidence, guide future research. This work supports the SSC priority of more research into the application of vasopressors in shock [23].

## Methods

This systematic review and its protocol were pre-registered on PROSPERO (CRD42023481778, 10th November 2023). The study was conducted in accordance with the PRISMA 2020 statement, elaboration and explanation [28, 29]. A PRISMA checklist is available in the additional files (see PRISMA Checklist). No financial support nor sponsorship was received.

### Inclusion criteria

This systematic review includes studies of adult patients (age ≥ 18 years) with septic shock, who by definition are receiving a vasopressor to maintain blood pressure. All iterations of the International Consensus Definitions for septic shock were used to capture the evolution of terminology [1, 30, 31]. In addition, study investigator defined septic shock was included to capture any studies pre-dating the first consensus definition. For inclusion, any study recruiting a mixed shock population required relevant renal outcomes specific to the septic sub-population to be reported.

Non-crossover randomised controlled trials comparing individual or combinations of vasopressors, including studies using a placebo comparator, were included. Cross-over trials and non-randomised studies of interventions were excluded as they were unable to address the objectives or risked introduction of unacceptable bias.

No limitations for study eligibility were imposed based on year of publication or language, however non-English papers where a translation was not available were excluded. Published peer reviewed study abstracts were included where possible. Studies with no original data, non-peer reviewed preprints and animal studies were excluded.

### Outcomes

Outcomes assessed were based on markers of short or long-term renal pathology resulting from the episode of septic shock. For inclusion, studies had to detail the primary outcome of AKI incidence of any severity, or one of the secondary outcomes. AKI was defined using any definition (Kidney Disease: Improving Global Outcomes (KDIGO), Acute Kidney Injury Network (AKIN) or Risk, Injury, Failure, Loss of End-stage kidney disease (RIFLE)) [32–34]. Secondary outcomes included: AKI duration, rate of RRT use, duration of RRT, renal failure free days, requirement for long term RRT and major adverse kidney events assessed at 30 and 90 days post AKI (MAKE 30 and MAKE 90) [35].

### Search strategy

A search strategy was developed using a Population, Intervention, Comparator, Outcome (PICO) approach utilising free-text keywords and subject headings for the concepts of “septic shock”, “vasopressor agents” and “renal impairment”. Searches were conducted separately on Medline and Embase (through Ovid) from inception to 4th March and Cochrane Central via the website on 7th March 2024. Where available, database-specific filters for controlled trials were applied. Full strategies are available in the additional files (see Supplementary Material, Tables S1-3). In addition, online abstracts of major congresses (Society of Critical Care Medicine, American Thoracic Society, International Symposium of Intensive Care and Emergency Medicine and European Society of Intensive Care Medicine) were searched manually for the previous 3 years (2020–23 or 2021–24). To identify any ongoing or unpublished trials, three registers were searched: Clinicaltrials.gov, WHO ICTRP and ISRCTN on 7th March 2024. Finally, reference and cited by lists from articles eligible for full-text review were manually screened. Database and trial registry searches were repeated on 13th March 2025 prior to final analysis.

### Selection of studies

All identified records were exported to Endnote 21 (Clarivate Plc, Philadelphia, USA) where duplicates were electronically and manually removed. Subsequently, one author (RM) screened all study titles, removing any that were clearly not eligible. Two authors (RM, MB) then independently screened study titles and abstracts. Studies that were deemed to be potentially eligible were retrieved in full to allow the same authors to independently assess for inclusion. Disagreement was resolved through discussion.

### Data extraction

Study data was extracted independently, and in duplicate, by two authors (RM, MB) using a standardised extraction form (see additional file Supplementary Material, Table S4). Where clarification was required on presented data, attempts were made to contact study authors.

Systematic review management was undertaken in Rayyan (Massachusetts, USA). Data was extracted on study design, methodology, interventions, controls, population demographics and outcomes relevant to this review. If study data was related to, but not reported in the form of, one of the review outcome measures, then attempts were made to convert and incorporate this data (for example creatinine level, urine output, study timeframes and AKI classification).

### Risk of bias assessment

Following pre-emptive standard setting, all included studies were assessed for risk of bias by two authors (RM, MB) independently and in duplicate using RoB 2 [36]. Studies were assessed both as a whole, but specifically in relation to the review outcomes reported. Disagreement was resolved through discussion. RoB 2 covers bias arising from: randomisation process, deviations from intended interventions, missing outcome data, measurement of the outcome and in selection of the reported results. Each domain was judged to be at low risk of bias, to have some concerns or be at high risk of bias. The overall risk of bias assessment was made for each study using domain scores and the significance of any concern. Publication biases were assessed through comparison of the trial registry search results and published final study reports.

### Data analysis

Due to renal outcomes often being secondary measures or documented only as part of the demographic description of study arms, a paucity of reported effect measures were found. Therefore, a narrative alongside tabular synthesis, analysis (including the calculation of odds ratios) and certainty assessment was performed. Studies were grouped by primary and secondary outcomes, and within this by vasopressor class and subsequently agent. Units for baseline clinical measures were converted to allow between study standardisation. In addition, weight-based vasopressor dosing was multiplied by an estimated weight of 70 kg for standardisation purposes.

## Results

### Literature search

Database and registry searches identified 3095 records. After duplicate removal, 2753 records were screened by title, then title and abstract, to highlight 31 reports for full text review. To these, 1 congress abstract was added. Subsequent searches of full text citations added a further 13 reports for review. Of the 45 reports sought for retrieval, 7 were trial registrations without published results and 1 report could not be obtained despite attempts to contact the author [37]. Ultimately, 37 reports underwent full text review.

**Fig. 1:**
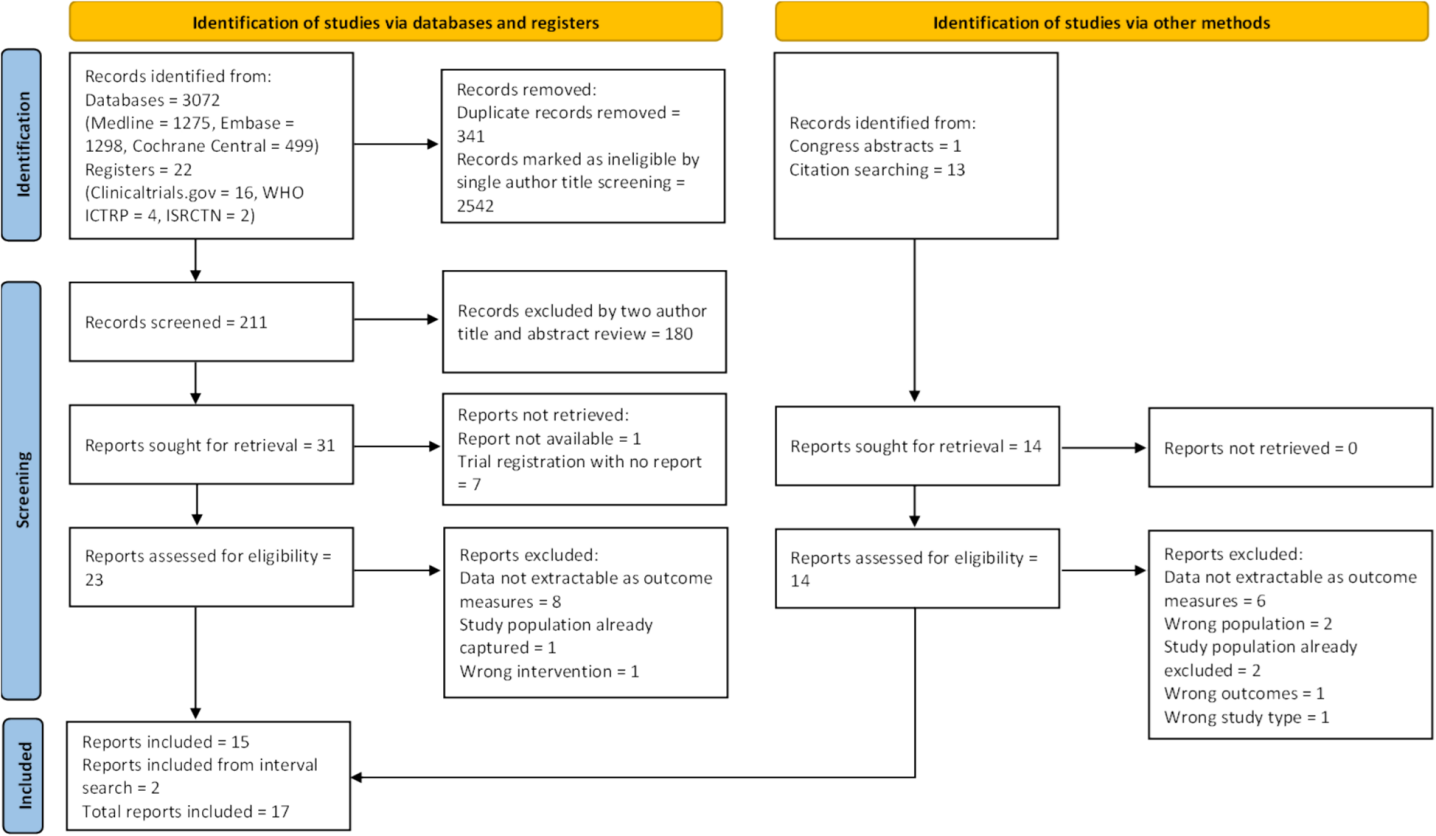
Prisma flow diagram of search results and study selection

Full text review excluded 22 reports. Study exclusion was most commonly due to reported renal measures, such as creatinine or urine output, that could not be converted into our primary or secondary outcomes (n = 14). Excluded study characteristics and reason for exclusion are detailed in the additional files (see Supplementary Material, Table S5). Two studies were subsequently added to the original 15 after interval searches. A full Prisma flow diagram of search results and study selection is presented in Fig. 1.

### Study characteristics

The 17 included studies were published between 2006 and 2025, incorporating 4259 septic shock patients. Most studies (65%) were single centre and either open-label or double-blinded randomised controlled trials (RCTs). Mean (± standard deviation, SD) participant age was 60.5 (± 7.1) years with 29.4% female representation. Study populations ranged in size from 20 to 1044. The majority of participants were admitted to intensive care units in Western Europe, North America, China or Australia. With one exception, no studies excluded patients with AKI at baseline [38]. In addition, fewer than half of reports detailed baseline renal dysfunction in the study population.

Septic shock was most commonly defined by the authors, often with reference to a SSC guideline. Thirteen of the 17 studies compared an antidiuretic hormone (ADH) analogue (vasopressin, terlipressin or selepressin) with standard care norepinephrine or placebo. Although norepinephrine was used in all studies, details of its formulation (base vs. salt) were only provided in one [39]. This is an important issue as the type of formulation can impact the actual effect of norepinephrine and therefore represents an important cause of heterogeneity [40]. Heterogeneity also existed across studies in terms of timing and duration of interventional vasopressor administration, as well as use of adjunctive therapies, including corticosteroids. Study primary outcome measures were heterogeneous and included 28-day mortality, change in vasopressor requirements, lactate clearance and perfusion measures. Only one study was designed with a renal primary outcome (kidney failure free days 28-days post randomisation) [41]. The characteristics of the 17 included studies are shown in Table 2.

**Table 2:** Included study characteristics, ordered by size

Study (year)	Design (centre)	Setting	n	Mean age	Population	I	Timing^∩^	Duration	C	Steroids	Primary outcome(s)
Zampieri et al. [54]* (2024)	Double-blinded RCT (MC)	Western Europe	1044		Mixed shock (62% septic)	D	< 4 h	Not fixed (median 17 days)	N	Allowed	28-day mortality
Laterre et al. [48]∞ (2019)	Double-blinded RCT (MC)	Western Europe & North America	828	66	Septic shock	S (N)	< 12 h	Not fixed (median 38 h)	P (N)	Not detailed	Ventilator- and vasopressor-free days
Russel et al. [51] (2008)	Double-blinded RCT (MC)	Australia & North America	778	61	Septic shock	V (N)	< 24 h	Not fixed	N	Allowed	28-day mortality
Liu et al. [44] (2018)	Double-blinded RCT (MC)	China	526	61	Septic shock	T (N)	Not detailed	Not fixed (mean 13 days)	N (N)	Not detailed	28-day mortality
Gordon et al. [41]† (2016)	Double-blinded RCT (MC)	United Kingdom	408	66	Septic shock	V (N)	< 6 h	Not fixed	N	Allowed^◊^	Kidney failure free days 28 days post randomisation
Hajjar et al. [46] (2019)	Double-blinded RCT (SC)	Brazil	244	63	Septic shock & cancer	V (N)	Not detailed	Not fixed (median 18 days)	N	Not detailed	28-day mortality
Choudhury et al. [53] (2017)	Open-label RCT (SC)	India	84	48	Septic shock & decompensated cirrhosis	T (N)	After 2 h IVF	Not fixed (median 24 h)	N	Allowed	MAP > 65 mmHg at 48 h
Gupta et al. [43] (2025)	Open-label RCT (SC)	India	65	46	Septic shock & acute on chronic liver failure	T (N)	Not detailed	Not detailed	N	Not allowed	MAP > 65 mmHg at 6 h
Sahoo et al. [52] (2022)	Open-label RCT (SC)	India	50	49	Septic shock	T (N)	Not detailed	12 h	N	Not detailed	Norepinephrine requirements
Morelli et al. [49] (2009)	Pilot RCT (SC)	Italy	45	67	Septic shock	VT (N)	Not detailed	48 h	N (N)	Allowed	Norepinephrine requirements
Morelli et al. [56] (2008)	Double-blinded RCT (SC)	Italy	32	70	Septic shock	Ph	0 h	12 h	N	Not detailed	Haemodynamic variables
Xiao et al. [45] (2016)	RCT (SC)	China	32	63	Septic shock	T (N)	Not detailed	6 h	N	Not detailed	Haemodynamic variables
Barzegar et al. [50] (2016)	Open-label RCT (SC)	Iran	30	64	Septic shock	V (N)	< 12 h of ICU admission	Not detailed	N	Allowed	Venous lactate & lactate clearance
Davoudi-Monfared et al. [47] (2021)	Pilot RCT (SC)	Iran	28	58	Septic shock	M (N)	< 24 h	5 days	N	Allowed	Lactate clearance
Lauzier et al. [39] (2006)	Open-label RCT (MC)	Canada & France	23	55	Septic shock	V (N)	< 12 h	48 h	N	Allowed	Haemodynamic variables & SOFA score
Wang et al. [38] (2022)	Pilot RCT (SC)	China	22	64	Septic shock	T (N)	Not detailed	24 h	N	Not detailed	Peak renal contrast enhanced ultrasound signal intensity
Chawla et al [42] (2014)	Pilot RCT (SC)	USA	20	63	Distributive shock (all septic)	A (N)	Not detailed	6 h	P (N)	Not detailed	Norepinephrine requirements

*RCT* Randomised controlled trial, *SC* single centre, *MC* multi centre, *n* number of participants, *I* intervention, *C* comparator, (*N*) use of open-label norepinephrine alongside interventional or study vasopressors, *D* dopamine, *V* vasopressin, *T* terlipressin, *A* angiotensin II, *Ph* phenylephrine, *S* selepressin, *M* midodrine, *N* norepinephrine, *IVF* intravenous fluid, *ICU* Intensive Care Unit, *P* placebo, *MAP* mean arterial pressure, *SOFA* sequential organ failure assessment

^*^Exploratory post-hoc analysis of SOAP II trial [55]. ∞ Adaptive design. † 2 × 2 factorial design with hydrocortisone or placebo. ^◊^ Excluded at baseline, but incorporated as part of factorial (2 × 2) study design. ^∩^ Timing of interventional vasopressor administration from shock onset, unless otherwise specified

### Primary outcome

The review primary outcome of AKI rate was reported in 8 (47%) of the included studies using the KDIGO or AKIN criteria or an undefined classification (see Table 3) [32, 33]. As both studies using an undefined AKI classification were more recent than the widely adopted KDIGO definition they were incorporated into the analysis [42, 43]. These 8 studies corresponded to a pool of 1345 patients with septic shock and mean age of 60 (± 6.4) years. Reported baseline characteristics included a lactate of 3.3 (± 1.4) mmol/L, Sequential Organ Failure Assessment (SOFA) score of 10.2 (± 3.5) and acute physiology and chronic health evaluation II (APACHE II) score of 24.8 (± 7.9). Unfortunately, due to granularity of the presented data, two of these studies precluded the accurate assessment of AKI rate amongst this population. The large trial by Liu et al. of terlipression against norepinephrine reported in prose that the study ‘failed to demonstrate a reduction in renal replacement therapy or acute kidney injury with terlipressin’. However, although AKI was defined using the KDIGO classification, no numeric rate is presented for each vasopressor arm [44]. The small study by Xiao et al., again of terlipressin against norepinephrine, presented results for urine output (in ml/kg/hr) over the 6 h study period that allowed conversion to KDIGO 1 AKI rates alongside the rate of ‘acute renal failure’ in both study arms [45]. When these studies are excluded, the rate of AKI amongst the remaining study population was 42%. This is likely an underestimate of overall AKI rate as the study by Gordon et al. reported only AKIN stage 3 renal injury [41]. In addition, the equivalence of the overall AKI and reported AKIN 3 rate may suggest that other studies also only focussed on more severe AKI without detailed differentiation between different AKI stages.

**Table 3:** Included studies reporting AKI primary outcome, grouped by interventional vasopressor

Study	Arms	n	Age	Dose*	MAP (mmHg)	HR (bpm)	Lactate (mmol/L)	SOFA	APACHE II	Creatinine (mg/dL)	AKI rate	OR [95% CI]	AKI criteria
Gordon et al. [41]	V (N)	204	67	< 0.06 IU/min	70	97	2.2		24	1.3	87 (42%)	0.82	AKIN (stage 3)
	N	204	65	< 12 mcg/min	69	98	2.4		24	1.5	97 (48%)	[0.55, 1.21]	
Hajjar et al. [46]	V (N)	121	64	0.04 IU/min	65	101	2.7	7		1.3	53 (42%)	1.06	AKIN
	N	123	62	24.5 mcg/min	64	100	2.8	7		1.3	52 (42%)	[0.64, 1.77]	
Liu et al. [44]	T (N)	260	61	1.3 mcg/min	68	118	4	11	19		No difference		KDIGO
	N (N)	266	61	12.4 mcg/min	68	118	3.8	12	19		No difference		
Gupta et al. [43]	T (N)	34	45	2.6–8.3 mcg/min	60	107				2.2	2 (6%)	0.60	
	N	31	48	7–35 mcg/min	58	104				2.1	3 (10%)	[0.07, 4.21]	
Xiao et al. [45]	T (N)	15	63	1.5 mcg/min			3.2		39		KDIGO 1 = 4 (27%) ARF = 2 (13%)	KDIGO 1 0.34 [0.07, 1.48]	KDIGO
	N	17	62				3.6		37		KDIGO 1 = 9 (53%) ARF = 6 (36%)	ARF 0.30 [0.04, 1.70]	
Wang et al. [38]	T (N)	10	62	1.5 mcg/min	91	88	2.1	9	19	0.8	2 (20%)	0.28	KDIGO
	N	12	66	5 mcg/min	89	85	1.5	8	23	0.9	6 (50%)	[0.03, 1.77]	
Chawla et al. [42]	A (N)	10	68	875 ng/min			4.6	15	27	1.9	10 (100%)		
	P (N)	10	57				7.1	17	34	2.8	8 (80%)		
Davoudi-Monfared et al. [47]	M (N)	15	58	10 mg 8 hrly			3.3	8	17	1.2	6 (40%)	1.06	KDIGO
	N	13	57	7.8 mcg/min			3.3	8	16	1.3	5 (38%)	[0.22, 5.22]	

*OR [95% CI]* odds ratio [95% confidence interval], (*N*) use of open-label norepinephrine alongside interventional or study vasopressors, *V* vasopressin, *T* terlipressin, *A* angiotensin II, *P* placebo, *M* midodrine, *N* norepinephrine (formulation unspecified), *MAP* mean arterial pressure, *HR* heart rate, *SOFA* sequential organ failure assessment, *APACHE II* Acute physiology and chronic health evaluation II score, *ARF* ‘Acute Renal Failure’ as reported by study authors, *KDIGO* Kidney Disease: Improving Global Outcomes, *AKIN* Acute Kidney Injury Network

^*^Standardised dose expressed as study mean or range, depending on source text reporting

ADH analogues were studied most extensively. Vasopressin was compared to norepinephrine in 2 studies, one of which demonstrated no benefit in overall AKI rate [46]. The second, by Gordon et al., was the only included study powered for renal outcomes. However, the observed reduction in severe renal injury (AKIN 3) with vasopressin, as compared to norepinephrine, failed to reach statistical significance (odds ratio [95% confidence interval] = 0.82 [0.55, 1.21]) [41]. Despite similar baseline clinical measures, these studies differed in both the doses of vasopressin (< 0.06 vs. 0.04 IU/min) and, perhaps more significantly, norepinephrine (< 12 vs. an average of 24.5 mcg/min). Potential heterogeneity also existed in exposure to corticosteroids, which were allowed as part of the factorial (2 × 2) design of the study by Gordon et al., but not reported by Hajjar et al. Also of note, the study by Hajjar et al., which showed no difference in AKI rate, recruited a sub-population of septic shock patients with solid organ malignancy. The inter-study heterogeneity from steroid therapy is unlikely to significantly impact the observed differences in AKI occurrence. This is due to the lack of benefit shown with controlled corticosteroid co-therapy on renal outcomes in the study by Gordon et al. The importance of the heterogeneity resulting from a specific septic shock population with cancer is difficult to evaluate. This is due to the lack of benefit demonstrated with vasopressin in the study by Hajjar et al., and high likelihood of inclusion of patients with cancer in any general septic shock population.

Terlipressin was compared to norepinephrine in 4 studies. Three studies, including a total of 119 participants, suggest improvement in the odds of AKI with terlipressin, however no study demonstrated statistical significance [38, 43, 45]. The dominant study of terlipressin (526 participants) reported no difference in the rate of KDIGO graded AKI between vasopressor arms [44]. Heterogeneity was present across all terlipressin studies, including in baseline clinical characteristics and vasopressor doses (terlipressin 1.3–8.3 mcg/min, norepinephrine 5–35 mcg/min). In addition, the study by Gupta et al. was of a sub-population of septic shock with acute on chronic liver failure [43]. Resulting heterogeneity is difficult to evaluate further, as was the case previously for septic shock subpopulation studies.

The remaining two studies were of a pilot nature. Chawla et al. recruited a sample of 20 particularly high risk patients with distributive shock, confirmed by the study author to all be septic, and compared angiotensin II to placebo [42]. Although designed to test for changes in open-label norepinephrine required to maintain target MAP, there was one new case of AKI giving 2 additional cases overall with angiotensin II as compared to placebo. In their work on enteral midodrine alongside standard care norepinephrine, Davoudi-Monfared et al. report one additional case of AKI with midodrine [47].

### Secondary outcomes

Secondary outcomes were reported in 13 (76%) studies, corresponding to 4120 patients with an average age of 61 (± 6.9) years. Outcomes captured were RRT rate (10 studies, 2430 patients), renal failure free days (2 studies, 1606 patients), duration of RRT (2 studies, 1236 patients) and AKI (1 study, 84 patients). Five studies included more than one of the primary or secondary outcomes. However, no studies reported on the requirement for long term RRT or MAKE30 and 90 (see Table 4).

**Table 4:** Included studies reporting secondary outcomes, grouped by interventional vasopressor

Study	Arms	n	Age	Dose*	MAP (mmHg)	HR (bpm)	Lactate (mmol/L)	SOFA	APACHE II	Creatinine (mg/dL)	RRT Rate	OR [95% CI]	Duration of AKI (days)	Duration of RRT (days)	Renal failure free days
Russel et al. [51]	V (N)	396	59	0.01–0.03 U/min	72		3.5		27						25 (6–28)
	N	382	62	5–15 mcg/min	73		3.5		27						23 (5–28)
Gordon et al. [41]	V (N)	204	67	< 0.06 IU/min	70	97	2.2		24	1.3	52 (25%)	0.63 [0.41, 0.96]		3 (2–7)	
	N	204	65	< 12 mcg/min	69	98	2.4		24	1.5	72 (35%)			3 (2–8)	
Hajjar et al. [46]	V (N)	121	64	0.04 IU/min	65	101	2.7	7		1.3	10 (8%)	0.57 [0.24, 1.28]			
	N	123	62	24.5 mcg/min	64	100	2.8	7		1.3	17 (14%)				
Morelli et al. [49]	V (N)	15	66	0.03 IU/min	53	100	3			2.2	5 (33%)	V 0.45 [0.04, 2.00]			
	T (N)	15	67	1.5 mcg/min	53	95	3.1			2.5	4 (27%)				
	N (N)	15	64	15 mcg/min	54	97	3.1			2.2	8 (53%)	T 0.33 [0.06, 1.53]			
Barzegar et al. [50]	V (N)	15	65	0.03 IU/min		90	2.3	12		1.3	4 (27%)	0.56 [0.11, 2.68]			
	N	15	63	9.8 mcg/min		87	2	12		1.4	6 (40%)				
Lauzier et al. [39]	V (NB)	13	51	0.11 IU/min	72	118	2.9		23		0				
	N	10	58	31 mcg/min	68	109	3.3		24		0				
Liu et al. [44]	T (N)	260	61	1.3 mcg/min	68	118	4	11	19		No difference				
	N (N)	266	61	12.4 mcg/min	68	118	3.8	12	19		No difference				
Choudhury et al. [53]	T (N)	42	47	2.9 mcg/min	61	105	3	14		2			< 3 days = 4/33 (12%) < 5 days = 6/16 (38%)		
	N	42	48	22.7 mcg/min	60	105	3	15		2			< 3 days = 4/32 (13%) < 5 days = 1/12 (8.3%)		
Sahoo et al. [52]	T (N)	25	49	1.4 mcg/min	58	96	4.4	9		1.1	1 (4%)	0.15 [0.01, 1.02]			
	N	25	49	26.2 mcg/min	59	98	4.2	9		1.2	6 (24%)				
Laterre et al. [48]	S (N)	562	67		70	102	2.7		26					2 ± 7.6	18 ± 14.3
	P (N)	266	66		69	101	2.6		26					1.6 ± 5.5	18.4 ± 13.9
Zampieri et al. [54]∞	D	542													
	N	502									D inferior to N				
Morelli et al. [56]	Ph	16	70								7 (44%)	4.96 [0.91, 43.2]			
	N	16	70								2 (13%)				
Davoudi-Monfared et al. [47]	M (N)	15	58	10 mg 8 hrly			3.3	8	17	1.2	2 (13%)	0.85 [0.08, 9.33]			
	N	13	57	7.8 mcg/min			3.3	8	16	1.3	2 (15%)				

*OR [95% CI] *odds ratio [95% confidence interval], (*N*) use of open-label norepinephrine alongside interventional or study vasopressors, *V* vasopressin, *T* terlipressin, *S* selepressin, *P* placebo, *D* dopamine, *Ph* phenylephrine, *M* midodrine, *N* norepinephrine (formulation unspecified), *NB* norepinephrine bitartrate, *MAP* mean arterial pressure, *HR* heart rate, *SOFA* sequential organ failure assessment, *APACHE II* Acute physiology and chronic health evaluation II score

^*^Standardised dose expressed as study mean or range, depending on source text reporting. ∞ Exploratory post-hoc analysis of SOAP II trial [55]

ADH analogues were again the most investigated vasopressors (vasopressin 5 studies, terlipressin 3 studies, vasopressin and terlipressin 1 study, selepressin 1 study). With the exception of the placebo controlled trial of selepressin by Laterre et al*.*, all studies used norepinephrine both in conjunction with the ADH analogue and as the comparator vasopressor [48].

When studying vasopressin, all trials except for the small study by Lauzier et al. report lower rates of RRT in the intervention arm [39, 41, 46, 49, 50]. However, this finding was only statistically significant in the study by Gordon et al. which, importantly, was powered for number of days alive and free of AKIN stage 3. The overall RRT rate with vasopressin and norepinephrine was 71 (19%) and 103 (28%) respectively. Of note, the studies reporting beneficial effects of vasopressin in terms of RRT rate administered a lower dose of vasopressin (all < 0.06 IU/min) [41, 46, 49, 50]. The single study using a higher average dose (0.11 IU/min) demonstrated no difference [39]. With regard to the other reported secondary outcomes, vasopressin therapy was associated with higher numbers of renal failure free days, but no difference in RRT duration [41, 51]. Baseline patient characteristics and illness scores were comparable between studies. However, as for the primary outcome, the study by Hajjar et al. recruited cancer patients with septic shock [46].

Two small studies of terlipressin report lower rates of RRT when compared to norepinephrine in septic shock [49, 52]. However, despite similar dosing, the dominant study by Lui et al. failed to show a difference between vasopressor arms [44]. Unfortunately, this outcome is documented in prose which precludes further evaluation. The remaining study of terlipressin uses higher doses and examines a sub-population of septic shock patients with cirrhosis [53]. This study suggests a lower duration AKI with terlipressin. However, caution is warranted in the interpretation of these results due to inconsistent denominator reporting.

Selepressin was studied in a single large adaptive trial by Laterre et al. designed for dose determination and efficacy evaluation [48]. The trial was stopped early, with 562 and 266 patients recruited to selepressin and norepinephrine arms respectively, due to futility. In addition to equivalence in the study's primary outcome of ventilator- and vasopressor-free days, no difference was demonstrated in either renal failure free days or duration of RRT.

The remaining three studies examined the adrenoceptor agonists dopamine, phenylephrine and midodrine. The study by Zampieri et al. is an exploratory post-hoc analysis of the SOAP II study [54, 55]. The original study recruited a mixed shock population, and although RRT free days was reported, results were not broken down into shock aetiology sub-populations. It was, therefore, screened out at full text review. However, this recent report contains novel statistical analysis of the existing data in the 542 (63%) of the study population with septic shock. Win ratio analysis demonstrates fewer wins with dopamine with regard to rate of RRT, suggesting this vasopressor was inferior to norepinephrine therapy in terms of this outcome. Phenylephrine, as fist line vasopressor, was reported by Morelli et al. from a small double blinded RCT [56]. This study demonstrated no benefit with phenylephrine, with higher rates of RRT (7, 44%) when compared to norepinephrine (2, 13%). Finally, a single study of midodrine demonstrated similar rates of RRT (2, 13% vs. 2, 15%) when this agent was given in addition to open label norepinephrine, as opposed to norepinephrine alone [47].

### Risk of bias and certainty assessments

**Fig. 2:**
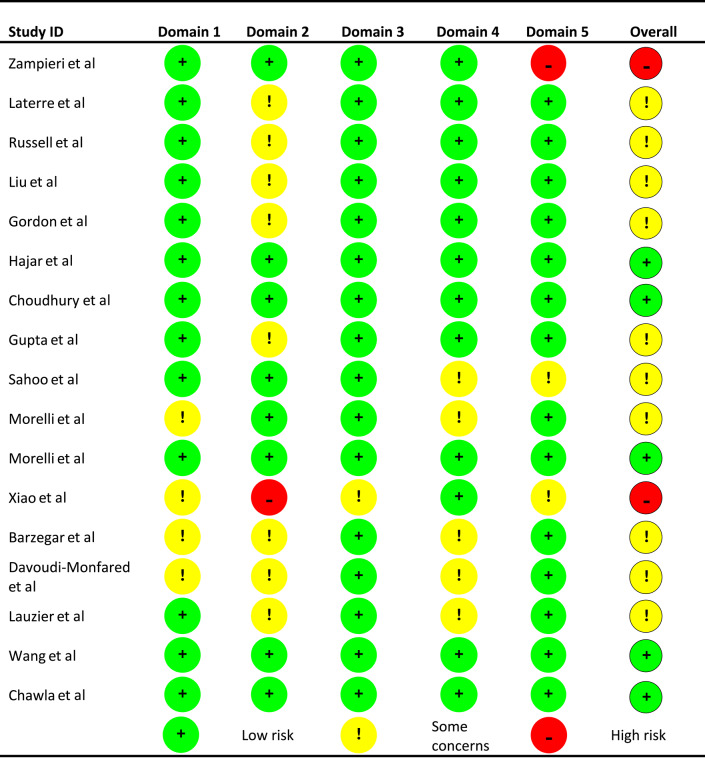
Traffic light plot for risk of bias in included studies. Domain 1 = Randomisation process, Domain 2 = Deviations from intended interventions, Domain 3 = Missing outcome data, Domain 4 = Measurement of the outcomes, Domain 5 = Selection of reported results

Most studies were deemed to have some concerns for bias overall. In the large RCTs bias concern was invariably due to analysis approach, whereby patients were excluded post randomisation [41, 44, 48, 51]. Another common concern for risk of bias was seen in the open-label studies using RRT rate as an outcome [39, 47, 49, 50, 52]. In these studies, although awareness of intervention allocation was judged as unlikely to influence measurement of RRT rate, there was concern of potential influence on a clinician’s decision to initiate therapy. Of note, two studies were deemed high risk of bias: Zampieri et al., due to its post-hoc nature, and Xiao et al. due to limited methodological reporting [45, 54]. A traffic light plot for the bias risks is presented in Fig. 2. It was concluded that risk of bias in individual or groups of studies did not significantly impact the results of this review. From database and trial registry searches, a number of unpublished studies were identified. However, these studies covered the full range of vasopressors with no clear trend suggestive of publication bias.

As specified in the methodology, all included studies were RCTs. However, these ranged from large multi-national double-blinded trials to small open-label and pilot studies. There was significant inter-study heterogeneity in terms of interventional and comparator vasopressor doses, timings and the use of additional therapies such as corticosteroids. This heterogeneity limits conclusions from study synthesis. In addition, as only one study was designed with a renal primary outcome, review findings based on the synthesis of study secondary outcomes should be viewed as exploratory and inform future research [41].

## Discussion

There are some important findings in this review that reflect limitations of evidence in this field. Firstly, most studies did not exclude patients with AKI. This undermines any findings related to ‘new AKI’ or ‘use of RRT’ as patients with and without existing injury were studied. It is also pertinent as there is a possibility that different vasopressors have differential effects in patients with and without AKI [57].

Secondly, RRT rate is a commonly used surrogate for recording renal injury. However, there are significant problems with this practice. Using RRT as the sole indicator of renal injury fails to capture patients with less severe AKI and, in a similar manner to reporting only AKIN 3 injury, potentially misses important benefits or harms that might be observed in these patients. This is despite this population still being at risk of adverse outcomes and chronic disease [58]. In addition, initiation of RRT means that a patient is, by default, classed as having KDIGO or AKIN stage 3 AKI [32, 33]. This is regardless of the indication, other renal measures or underlying organ function. However, it is widely accepted that great variation exists in RRT practice between both clinicians and centres [59]. Therefore, reliance on this outcome measure risks the introduction of further heterogeneity into study results. Heterogenous practice in terms of fluid management and blood pressure targets, which are not standardised, further confounds these trials.

Trials of vasopressors in septic shock are limited by the lack of a standardised set of reported renal outcomes. In addition to RRT rate, current practice is often to report average creatinine values or urine outputs. AKI rate reporting is surprisingly uncommon, and is occasionally also performed using an undefined classification. Although debated due to differing definitions, the composite MAKE outcomes are unused in these trials [60]. In this review, outcome reporting practice meant that many studies were excluded as renal measures could not be converted to our primary or secondary outcomes. Alongside heterogeneity, it also prevented us from undertaking a meta-analysis and developing forest plots of the data. Although renal endpoints are controversial, utilisation of a selection of recommended renal outcomes for shock trials would aid both study comparison and synthesis of literature in this field [61, 62]. This approach has already been proposed for perioperative studies [63].

A further limitation results from the lack of renal focussed, and therefore powered, studies in septic shock. Apart from the VANISH trial by Gordon et al., all studies included in this review were not powered for renal outcomes [41]. In addition, given our strict eligibility criteria, many studies could not be included as they focussed on mixed shock populations or did not report renal outcomes specific to the septic sub-population. This resulted in the exclusion of a number of large studies, and specifically the ATHOS 3 trial, a multi-national RCT exploring the role of angiotensin II [64]. Although the ATHOS 3 trial recruited a mixed vasodilatory shock population, the majority of whom were septic, renal data for our outcomes was not extractable for the whole or sub-population of interest. This finding was confirmed following direct communication with the authors of the ATHOS 3 trial. This is unfortunate given post-hoc analysis suggests improvement in liberation from RRT with angiotensin II as compared to standard care [65]. Although it is appreciated that exact identification of shock aetiology can be problematic, and significant overlap exists, description of study sub-populations as well as their outcome data is needed in trials to enhance available data from mixed shock trials for focused reviews such as this.

Overall, our review demonstrates the lack of strong evidence for improved renal outcomes in septic shock when a particular vasopressor is used. Table 5 summarises this finding across all vasopressors and outcomes. In terms of AKI rate, only the ADH analogues vasopressin and terlipressin have been extensively studied, with neither agent demonstrating benefit over norepinephrine. The exceptions are trials with small sample sizes or when limited to AKIN AKI stage 3, but even then significance is not reached. Results for a reduction in the problematic outcome of RRT rate are perhaps more promising, with a trend towards reduction in RRT requirement with vasopressin. However, significance is rarely achieved and, compared to the conclusions from two published systematic reviews focussed on renal outcome in mixed shock, our results are less favourable for the non-catecholamine vasopressors [21, 22]. There are a number of possible reasons for this. Primarily, other reviews include heterogeneous shock populations and non-randomised studies of interventions. Considering the argument that AKI is a collection of syndromes, the different result seen here as opposed to more diverse populations may be secondary to benefit within particular shock associated AKI states [66].

**Table 5:** Summary of results and evidence

Outcome	Vasopressor	Studies	RCT types	N	Result	Inter-study heterogeneity	Evidence base	Overall risk of bias	Comments
AKI rate	V	2 [41, 46]	2 double blinded	652	No benefit	Moderate	Limited	Some concern	Includes only study powered for renal outcomes
	T	4 [38, 43–45]	1 double blinded, 1 open label, 1 not detailed, 1 pilot	645	No benefit	High	Limited	Some concern	
	A	1 [42]	Pilot	20	Possible harm		Very limited	Low risk	
	M	1 [47]	Pilot	28	Possible harm		Very limited	Some concern	
AKI duration	T	1 [53]	Open label	84	Possible benefit		Very limited	Low risk	Caution warranted in interpretation due to reporting
RRT rate	V	5 [39, 41, 46, 49, 50]	2 double blinded, 2 open label, 1 pilot	735	Possible benefit	Moderate	Limited	Some concern	Includes only study powered for renal outcomes
	T	3 [44, 49, 52]	1 pilot, 1 double blinded, 1 open label	606	No benefit	Moderate	Limited	Some concern	
	D	1 [54]	Double blinded*	1044	Possible harm		Limited	High risk	
	Ph	1 [56]	Double blinded	32	Possible harm		Very limited	Low risk	
	M	1 [47]	Pilot	28	No benefit		Very limited	Some concern	
RRT duration	V	1 [41]	Double blinded	408	No benefit		Very limited	Some concern	Includes only study powered for renal outcomes
	S	1 [48]	Double blinded	828	No benefit		Very limited	Some concern	
Renal failure free days	V	1 [51]	Double blinded	778	No benefit		Very limited	Some concern	
	S	1 [48]	Double blinded	1064	No benefit		Very limited	Some concern	
Long term RRT		0					No evidence		
MAKE 30		0					No evidence		
MAKE 90		0					No evidence		

*N* number of individuals, *AKI* acute kidney injury, *RRT* renal replacement therapy, *MAKE* 30/90 major adverse kidney events assessed at 30 and 90 days, *V* vasopressin, *T* terlipressin, *A* angiotensin II, *M* midodrine, *D* dopamine, *Ph* phenylephrine, *S* selepressin

^*^ Exploratory post-hoc analysis of SOAP II trial [55]

A final, but important, consideration is that our results may also be limited by study population heterogeneity. Similar to the issues with mixed shock reviews, the patients included in this study may also be too diverse. Septic shock cohorts are likely to be composed of many sub-populations, each with different phenotypes, chronic diseases, risk factors and exposures to certain medications. This is in addition to the between study heterogeneity in norepinephrine base doses, timing and duration of interventional vasopressors as well as co-therapy use. It is possible that certain septic shock sub-groups may have renal benefit from particular vasopressors, but that this signal is lost in both individual studies and this review [57].

## Conclusion

Vasopressor therapy is fundamental to the management of septic shock, and this condition is commonly complicated by AKI. Individual and classes of vasopressors have differing pharmacodynamic effects on the renal vasculature and perfusion. However, vasopressor studies reporting renal outcomes in this patient population yield limited and heterogeneous data. This results in a paucity of evidence for an effect of vasopressor choice on renal outcomes. Future researchers should focus not only on large, appropriately powered, RCTs of vasopressor effect on standardised renal outcomes, but also on smaller, more targeted trials where specific or enhanced sub-populations of septic shock patients are given specific vasopressors at particular doses and their renal outcomes explored.

## Supplementary Information

Supplementary Material 1. Full search strategies for Medline, Embase and Cochrane Central, Standardised data extraction form, Excluded study characteristics and exclusion rationale

Supplementary Material 2. PRISMA checklist for systematic review reporting

## Data Availability

All data generated or analysed during this study are included in this published article [and its supplementary information files].

## Abbreviations

AKI: Acute kidney injury
RRT: Renal replacement therapy
MAKE: Major adverse kidney events
RAS: Renin-angiotensin system
SSC: Surviving sepsis campaign
MAP: Mean arterial pressure
PICO: Population, intervention, comparator, outcome
RCT: Randomised controlled trial
SD: Standard deviation
ADH: Antidiuretic hormone
KDIGO: Kidney disease: improving global outcomes
AKIN: Acute kidney injury network
SOFA: Sequential organ failure assessment
APACHE II: Acute physiology and chronic health evaluation II score
