# Supplementary material for: The effects of vasopressor choice on renal outcomes in septic shock: a systematic review of randomised trials as a guide for future research
Source: Crit Care. 2025 Oct 2;29:417. doi: 10.1186/s13054-025-05573-7 (PMC12492594; doi:10.1186/s13054-025-05573-7)
Supplement: Supplementary file 1 — Supplementary Material 1. Full search strategies for Medline, Embase and Cochrane Central, Standardised data extraction form, Excluded study characteristics and exclusion rationale [file 13054_2025_5573_MOESM1_ESM.docx]

**The effects of vasopressor choice on renal outcomes in septic shock: A systematic review of randomised trials as a guide for future research.**

Rory McDonald^1,2,3*^

Michael Burns^4^

Adrian Wong^1,2,3^

Carolyn Smith^5^

Marlies Ostermann^1,6^

Sam Hutchings^1,2,3^

1. Department of Inflammation Biology, School of Immunology and Microbial Sciences, King’s College London, London, UK

2. Academic Department of Anaesthesia and Critical Care, Royal Centre for Defence Medicine, Birmingham, UK

3. Department of Critical Care, King’s College Hospital, King’s College Hospital NHS Foundation Trust, London, UK

4. Department of Critical Care, Bristol Royal Infirmary, University Hospitals Bristol and Weston NHS Foundation Trust, Bristol, UK

5. Bodleian Health Care Libraries, University of Oxford, UK

6. Department of Critical Care, St Thomas’ Hospital, Guy’s and St Thomas’ NHS Foundation Trust, London, UK

*Correspondence: rory.mcdonald@nhs.net

**Supplementary Material**

| 1 | sepsis/ or exp bacteremia/ or exp fungemia/ or shock, septic/ or Systemic Inflammatory Response Syndrome/ |
| --- | --- |
| 2 | (((sepsis or septic*) adj3 shock) or bacteremia or fungemia or Systemic Inflammatory Response Syndrome or SIRS).ti,ab,kf. |
| 3 | 1 or 2 |
| 4 | renal insufficiency/ or exp acute kidney injury/ or renal replacement therapy/ or continuous renal replacement therapy/ or hemofiltration/ or hemoperfusion/ or hybrid renal replacement therapy/ or intermittent renal replacement therapy/ or hemodiafiltration/ or peritoneal dialysis/ or Creatinine/ or anuria/ or oliguria/ or urine/ |
| 5 | (((renal or kidney*) adj4 (insufficient* or injur* or failure* or dysfunction* or impair* or disease*)) or (renal adj3 replacement) or RRT or hemofiltra* or haemofiltra* or hemodia* or haemodia* or dialysis or creatinine or (urine adj3 output) or oliguria or anuria or oligoanuria).ti,ab,kf. |
| 6 | 4 or 5 |
| 7 | vasoconstrictor agents/ or angiotensin ii/ or arginine vasopressin/ or ephedrine/ or epinephrine/ or metaraminol/ or midodrine/ or norepinephrine/ or phenylephrine/ or terlipressin/ or vasopressins/ or dopamine/ or methylene blue/ |
| 8 | (vasoconstrictor* or vasopressor* or angiotensin* or vasopressin* or anti diuretic hormone or ADH or AVP or ephedrine or epinephrine or adrenaline or metaraminol or midodrine or norepinephrine or noradrenaline or phenylephrine or terlipressin or dopamine or methylene blue).ti,ab,kf. |
| 9 | 7 or 8 |
| 10 | 3 and 6 and 9 |

**Table S1** Medline search strategy.

| 1 | sepsis/ or systemic inflammatory response syndrome/ or exp bacteremia/ or exp fungemia/ or exp septic shock/ or exp septicemia/ |
| --- | --- |
| 2 | (((sepsis or septic*) adj3 shock) or bacteremia or fungemia or Systemic Inflammatory Response Syndrome or SIRS).ti,ab,kf. |
| 3 | 1 or 2 |
| 4 | kidney failure/ or acute kidney failure/ or anuria/ or exp kidney tubule necrosis/ or mild renal impairment/ or moderate renal impairment/ or oliguria/ or renal replacement therapy-dependent renal disease/ or severe renal impairment/ or subclinical renal impairment/ or renal replacement therapy/ or exp continuous renal replacement therapy/ or exp hemodiafiltration/ or exp hemodialysis/ or exp hemofiltration/ or intermittent renal replacement therapy/ or exp peritoneal dialysis/ or creatinine clearance/ or creatinine blood level/ or urine/ |
| 5 | (((renal or kidney*) adj4 (insufficient* or injur* or failure* or dysfunction* or impair* or disease*)) or (renal adj3 replacement) or RRT or hemofiltra* or haemofiltra* or hemodia* or haemodia* or dialysis or creatinine or (urine adj3 output) or oliguria or anuria or oligoanuria).ti,ab,kf. |
| 6 | 4 or 5 |
| 7 | vasoconstrictor agent/ or angiotensin ii/ or argipressin/ or midodrine/ or noradrenalin/ or norephedrine/ or terlipressin/ or vasopressin/ or epinephrine/ or ephedrine/ or metaraminol/ or phenylephrine/ or dopamine/ or methylene blue/ |
| 8 | (vasoconstrictor* or vasopressor* or angiotensin* or vasopressin* or anti diuretic hormone or ADH or AVP or ephedrine or epinephrine or adrenaline or metaraminol or midodrine or norepinephrine or noradrenaline or phenylephrine or terlipressin or dopamine or methylene blue).ti,ab,kf. |
| 9 | 7 or 8 |
| 10 | 3 and 6 and 9 |
| 11 | limit 10 to ("remove medline records" and embase) |

**Table S2** Embase search strategy.

| ID | Search |
| --- | --- |
| #1 | MeSH descriptor: [Sepsis] this term only |
| #2 | MeSH descriptor: [Bacteremia] explode all trees |
| #3 | MeSH descriptor: [Fungemia] explode all trees |
| #4 | MeSH descriptor: [Shock, Septic] this term only |
| #5 | MeSH descriptor: [Systemic Inflammatory Response Syndrome] this term only |
| #6 | (((sepsis or septic) NEAR/3 shock) or bacteremia or fungemia or systemic inflammatory response syndrome or SIRS) |
| #7 | #1 OR #2 OR #3 OR #4 OR #5 OR #6 |
| #8 | MeSH descriptor: [Renal Insufficiency] this term only |
| #9 | MeSH descriptor: [Acute Kidney Injury] explode all trees |
| #10 | MeSH descriptor: [undefined] explode all trees |
| #11 | MeSH descriptor: [Continuous Renal Replacement Therapy] this term only |
| #12 | MeSH descriptor: [Hemofiltration] this term only |
| #13 | MeSH descriptor: [Hemoperfusion] this term only |
| #14 | MeSH descriptor: [Hybrid Renal Replacement Therapy] this term only |
| #15 | MeSH descriptor: [Intermittent Renal Replacement Therapy] this term only |
| #16 | MeSH descriptor: [Hemodiafiltration] this term only |
| #17 | MeSH descriptor: [Peritoneal Dialysis] this term only |
| #18 | MeSH descriptor: [undefined] explode all trees |
| #19 | MeSH descriptor: [Anuria] this term only |
| #20 | MeSH descriptor: [Oliguria] this term only |
| #21 | MeSH descriptor: [Urine] explode all trees |
| #22 | (((renal or kidney*) NEAR/4 (insufficient* or injur* or failure* or dysfunction* or impair* or disease*)) or (renal NEAR/3 replacement) or RRT or hemofiltra* or haemofiltra* or hemodia* or haemodia* or dialysis or creatinine or (urine NEAR/3 output) or oliguria or anuria or oligoanuria) |
| #23 | #8 OR #9 OR #10 OR #11 OR #12 OR #13 OR #14 OR #15 OR #16 OR #17 OR #18 OR #19 OR #20 OR #21 OR #22 |
| #24 | MeSH descriptor: [Vasoconstrictor Agents] this term only |
| #25 | MeSH descriptor: [Angiotensin II] this term only |
| #26 | MeSH descriptor: [Arginine Vasopressin] this term only |
| #27 | MeSH descriptor: [Ephedrine] this term only |
| #28 | MeSH descriptor: [Epinephrine] this term only |
| #29 | MeSH descriptor: [Metaraminol] this term only |
| #30 | MeSH descriptor: [Midodrine] this term only |
| #31 | MeSH descriptor: [Norepinephrine] this term only |
| #32 | MeSH descriptor: [Phenylephrine] this term only |
| #33 | MeSH descriptor: [Terlipressin] this term only |
| #34 | MeSH descriptor: [Vasopressins] this term only |
| #35 | MeSH descriptor: [Dopamine] this term only |
| #36 | MeSH descriptor: [Methylene Blue] this term only |
| #37 | (vasoconstrictor* or vasopressor* or angiotensin* or vasopressin* or anti diuretic hormone or ADH or AVP or ephedrine or epinephrine or adrenaline or metaraminol or midodrine or norepinephrine or noradrenaline or phenylephrine or terlipressin or dopamine or methylene blue) |
| #38 | #24 OR #25 OR #26 OR #27 OR #28 OR #29 OR #30 OR #31 OR #32 OR #33 OR #34 OR #35 OR #36 OR #37 |
| #39 | #7 AND #23 AND #38 |

**Table S3** Cochrane Central search strategy.

| **General** | | |
| --- | --- | --- |
| Study Title |  | |
| First author (Surname, initial) |  | |
| Date of publication |  | |
| Journal |  | |
| Congress abstract |  | |
| Authors contacted |  | |
| Published protocol |  | |
| Trial registration |  | |
| Study type |  | |
| Population |  | |
| Intervention |  | |
| Control/comparator |  | |
| Outcome (primary) |  | |
| *Comments* |  | |
| **Population** | | |
| Sepsis definition |  | |
| Shock definition |  | |
| Inclusion criteria |  | |
| Exclusion criteria |  | |
| Time to study recruitment |  | |
| Prior vasopressor use |  | |
| AKI definition |  | |
| *Comments* |  | |
| **Intervention** | | |
| Intervention vasopressor |  | |
| Dose schedule |  | |
| Dose |  | |
| Duration |  | |
| Comparator vasopressor |  | |
| Dose schedule |  | |
| Dose |  | |
| Duration |  | |
| Additional vasopressors |  | |
| Dose |  | |
| Duration |  | |
| Adjunct therapies |  | |
| *Comments* |  | |
|  | **Intervention** | **Control** |
| Participant number |  |  |
| Gender (number male) |  |  |
| Age |  |  |
| Geographic region |  |  |
| *Comments* |  | |
| APACHE II |  |  |
| SOFA |  |  |
| Lactate (baseline) |  |  |
| Heart rate (baseline) |  |  |
| MAP (baseline) |  |  |
| Creatinine (baseline) |  |  |
| AKI/renal dysfunction (baseline) |  |  |
| *Comments* |  | |
| Creatinine (maximum) |  |  |
| Urine output |  |  |
| AKI rate |  |  |
| AKI severity |  |  |
| AKI duration |  |  |
| RRT rate |  |  |
| RRT duration |  |  |
| RRT free days |  |  |
| Rate of ESRF |  |  |
| MAKE30 rate (& breakdown) |  |  |
| MAKE90 rate (& breakdown) |  |  |
| *Comments* |  | |

**Table S4** Standardised data extraction form

| **Study (year)** | **Design (Centre)** | **Setting** | | **n** | | | **Mean Age** | | **Population** | | | **I** | | | **C** | | | **Primary Outcome** | | **Exclusion Reason** |
| --- | --- | --- | --- | --- | --- | --- | --- | --- | --- | --- | --- | --- | --- | --- | --- | --- | --- | --- | --- | --- |
| Albanèse *et al*  (2005) | Open-label RCT  (SC) | France | 20 | | | 66 | | Septic shock | | | N | | | T | | | Haemodynamic variables & oxygen delivery/consumption | | | Unusable renal measures |
| Hu *et al*  (2023) | RCT (SC) | China | | 80 | | | 51 | | Septic shock | | | N | | | D | | | Haemodynamic variables & 'renal function' | | Unusable renal measures |
| Hussain *et al*  (2014) | RCT (SC) | Pakistan | | 42 | | | 48 | | Septic shock | | | P | | | N | | | Haemodynamic variables | | Unusable renal measures |
| Hamden *et al*  (2009) | Double-blinded RCT  (SC) | Egypt | 16 | | | 40 | | Septic shock & burns | | | N | | | V | | | Haemodynamic variables, biochemistry & urine output | | | Unusable renal measures |
| Kirov *et al*  (2001) | Pilot RCT (SC) | Russia | | 20 | | | 57 | | Septic shock | | | MB | | | P | | | Haemodynamic variables, organ function & 28 day mortality | | Unusable renal measures |
| Mathur *et al*  (2007) | RCT (SC) | India | | 50 | | | 54 | | Septic shock | | | D | | | N | | | Haemodynamic variables | | Unusable renal measures |
| Patel *et al*  (2002) | Double-blinded RCT  (MC) | Canada | 24 | | | 68 | | Septic shock | | | V | | | N | | | Noradrenaline requirements | | | Unusable renal measures |
| Ibarra-Estrada *et al* (2023) | Double-blinded RCT  (SC) | Mexico | | 91 | | | 47 | | Septic shock | | | MB | | | P | | | Time to shock resolution | | Unusable renal measures |
| Patro *et al*  (2021) | Open-label RCT  (SC) | India | 50 | | | 36 | | Septic shock | | | V | | | Ph | | | Haemodynamic variables, biochemistry & urine output | | | Unusable renal measures |
| Martin *et al*  (1993) | Double-blinded RCT  (SC) | France∞ | 32 | | | 53 | | Septic shock | | | D | | | N | | | Haemodynamic variables & oxygen delivery/consumption | | | Unusable renal measures |
| Malay *et al*  (1999) | Double-blinded RCT  (SC) | USA | 10 | | | 55 | | Septic shock | | | V | | | P | | | Haemodynamic variables | | | Unusable renal measures |
| Memis *et al*  (2002) | Double-blinded RCT  (SC) | Turkey∞ | 30 | | | 51 | | Septic shock | | | MB | | | P | | | Plasma cytokine concentrations | | | Unusable renal measures |
| Jain *et al*  (2010) | RCT (SC) | India∞ | | 54 | | | 44 | | Septic shock | | | N | | | Ph | | | Haemodynamic variables & oxygen delivery | | Unusable renal measures |
| Russell *et al*  (2017) | Double-blinded RCT  (MC) | Western Europe  & North America | | 52 | | | 63 | | Septic shock | | | S | | | P | | | Haemodynamic variables and noradrenaline requirements | | Unusable renal measures |
| De Backer *et al*  (2010) | RCT (MC) | Western Europe | | 1044* | | | 68† | | Mixed shock | | | D | | | N | | | 28-day mortality | | Mixed shock population |
| Khanna *et al*  (2017) | Double-blinded RCT  (MC) | North America, Europe  & Australasia | | 259* | | | 64† | | Vasodilatory shock | | | A | | | P | | | MAP response at 3 hrs | | Mixed shock population |
| Tumlin *et al*  (2018) | Post-hoc analysis  (Khanna *et al*) | | | |  | |  | | |  | | |  | | |  | | |  | Previously excluded study population |
| Zarbock *et al*  (2023) | Post-hoc analysis  (Khanna *et al*) | | | |  | |  | | |  | | |  | | |  | | |  | Previously excluded study population |
| Myburgh *et al*  (2008) | Double-blinded RCT  (MC) | Australia | | 158* | | | 60† | | Mixed shock^◊^ | | | Adr | | | N | | | Time to achieving target MAP off vasopressors | | No renal outcomes |
| Hammond *et al*  (2018) | Open-label trial (SC) | USA | | 82 | | | 61 | | Septic shock | | | N | | | V | | | Time to achieve MAP | | Wrong study type |
| Schmoelz *et a*l  (2006) | Double-blinded RCT  (SC) | Germany∞ | 41 | | | 53 | | Septic shock | | | Do | | | D | | |  | | | Wrong intervention |
| Gordon *et al*  (2010) | Post-hoc analysis  (Russel *et al*) | | | |  | |  | | |  | | |  | | |  | | |  | Study population already captured |

*∞ Assumed location based on author affiliations. * Septic shock sample size (extracted from overall study population). † Total study shock population. ^◊^ Septic shock subpopulation analysed separately.*

**Table S5** Excluded study characteristics and exclusion rationale. RCT = Randomised controlled trial, SC = single centre, MC = multi centre, n = number of participants, I = intervention, C = comparator, N = noradrenaline, T = terlipressin, D = dopamine, P = placebo, V = vasopressin, MB = methylene blue, Ph = phenylephrine, S = selepressin, A = angiotensin II, Adr = adrenaline, Do = Dopexamine.
